
JOURNAL INFORMATION
==============================
NLM Title Abbreviation: ScientificWorldJournal
Journal ID: ScientificWorldJournal
NLM Journal ID: 101131163
Journal ID: TSWJ

The Scientific World Journal

ISSN: 2356-6140
EISSN: 1537-744X
Publisher: Wiley

ARTICLE INFORMATION
==============================
PMCID: PMC6009264
PMID: 29973836
DOI: 10.1100/tsw.2002.61
Article ID: 323787
Article version: 1
Subjects: Short Report

Molecular Breeding of Genes, Pathways and Genomes by Dna Shuffling

Stemmer WIllem P.C. *
Maxygen Inc., Redwood City, CA 940663, USA
*WIllem P.C. Stemmer: pim_stemmer@maxygen.com
Publication date: 2002
Electronic publication date: 2002 Apr 20
Volume: 2
First page: 130
Last page: 131
Copyright: Copyright © 2002 WIllem P.C. Stemmer.
Copyright year: 2002
License: This is an open access article distributed under the Creative Commons Attribution License, which permits unrestricted use, distribution, and reproduction in any medium, provided the original work is properly cited.
License URL: https://creativecommons.org/licenses/by/3.0/

==============================
Advances in Gene Technology: The Genome and Beyond –
Structural Biology for Medicine (Proceedings of the 2002
Miami *Nature Biotechnology* Winter Symposium)
*TheScientificWorld* 2002, 2(S2), 130–131
ISSN 1532-2246; DOI 10.1100/tsw.2002.61

### MOLECULAR BREEDING OF GENES, PATHWAYS AND GENOMES BY DNA SHUFFLING

Willem P.C. Stemmer
VP R&D, Maxygen, Inc., 515 Galveston Drive, Redwood City, CA 94063
pim_stemmer@maxygen.com; www.maxygen.com

Genomics is yielding a wealth of sequence and functional information that impacts all areas of biotechnology, including pharmaceuticals, vaccines, chemicals/enzymes, and agriculture. However, developing products from these novel genes and pathways typically requires optimization of the gene(s) for the commercially relevant property. Maxygen uses its Molecular Breeding technology to optimize human genes for improved performance as protein pharmaceuticals; typical goals are increasing potency, reducing side effects and modulating specificity and half-life. We also improve the effectiveness of vaccines by breeding of related pathogen sequences. In the chemicals area, we breed genes encoding enzymes to yield better enzymes for a wide range of industrial and biosynthesis applications. We also breed multigene pathways and whole microbial genomes, resulting in microbes with improved fermentation properties. In agriculture, we have used molecular breeding to evolve genes with improved insect resistance and other properties of agricultural interest. In all of these examples functional optimization of the gene(s) was essential. Molecular breeding typically starts within a variety of sequences from natural diversity, such as the same gene from related species. This pool of starting genes is then shuffled by (mostly) homologous recombination, resulting in a large pool of chimeric genes, which is assayed for improved properties in a variety of functional screens. The libraries of chimeric genes that are created by molecular breeding have surprisingly high average functionality but also have a wide range of combinations of properties ranging well beyond that of the parental molecules. Screening of a relatively small number of clones (in some cases only few hundred) from such libraries has yielded commercially important improvements. The small number of screens required to obtain improvements allows us to screen directly in complex assays which closely mimick the final commercial application. We feel that the quality of the screen is essential for successful product development, more so then the number of clones screened. In turn, the quality of the library is critical for successfully using the highest quality screens, which are typically low throughput. When possible, we will follow a lower quality, high throughput screen with a low throughput, high quality screen. I will describe a broad range of examples of genes, pathways and genomes that have been improved by this approach.

### REFERENCES

1. Raillard, S., Krebber, A., Chen, Y., Ness, J.E., Bermudez, E., Trinidad, R.,

Fullem, R., Davis, C., Welch, M., Seffernick, J., Wackett, L.P., Stemmer, W.P.C., and Minshull, J. (2001) Chem. Biol. 125, 1–9.

2. Ness et al. (2000) Molecular Breeding: the natural approach to protein design. In Evolutionary Approaches to Protein Design. Arnold, F., Ed. Advances in Protein Chemistry, Vol. 55. Academic Press, San Diego. pp 261–292.
3. Minshull, J. and Stemmer, W.P.C. (1999) Curr. Opin. Chem. Technol. 3, 284–290.
4. Chang et al. (1999) Nat. Biotechnol. 17, 793–797.
5. Ness et al. (1999) Nat. Biotechnol. 17, 893–896.
6. Crameri, A., Raillard, S.-A., Bermudez, E., and Stemmer, W.P.C. (1998) Nature 391, 288–291.
7. Patten, P.A., Howard, R.J., and Stemmer, W.P.C. (1997) Curr. Opin. Biotechnol. 8, 724–733.
8. Stemmer, W.P.C. (1995) Bio/Technology 13, 549–553.
9. Stemmer, W.P.C. (1994) Nature 370, 389–391.
